# MATIN: A Random Network Coding Based Framework for High Quality Peer-to-Peer Live Video Streaming

**DOI:** 10.1371/journal.pone.0069844

**Published:** 2013-08-05

**Authors:** Behrang Barekatain, Dariush Khezrimotlagh, Mohd Aizaini Maarof, Hamid Reza Ghaeini, Shaharuddin Salleh, Alfonso Ariza Quintana, Behzad Akbari, Alicia Triviño Cabrera

**Affiliations:** 1 Faculty of Computing, Universiti Teknologi Malaysia, Johor Bahru, Malaysia; 2 Department of Mathematics, Faculty of Science, Universiti Teknologi Malaysia, Johor Bahru, Malaysia; 3 Faculty of Electrical and Computer Engineering, Tarbiat Modares University, Tehran, Iran; 4 Departamento Tecnología Electrónica, E.T.S.I, Telecommunication, University of Malaga, Malaga, Spain; 5 Escuela de Ingenierías, Departamento Ingeniería Eléctrica, University of Malaga, Malaga, Spain; University of Warwick, United Kingdom

## Abstract

In recent years, Random Network Coding (RNC) has emerged as a promising solution for efficient Peer-to-Peer (P2P) video multicasting over the Internet. This probably refers to this fact that RNC noticeably increases the error resiliency and throughput of the network. However, high transmission overhead arising from sending large coefficients vector as header has been the most important challenge of the RNC. Moreover, due to employing the Gauss-Jordan elimination method, considerable computational complexity can be imposed on peers in decoding the encoded blocks and checking linear dependency among the coefficients vectors. In order to address these challenges, this study introduces MATIN which is a random network coding based framework for efficient P2P video streaming. The MATIN includes a novel coefficients matrix generation method so that there is no linear dependency in the generated coefficients matrix. Using the proposed framework, each peer encapsulates one instead of *n* coefficients entries into the generated encoded packet which results in very low transmission overhead. It is also possible to obtain the inverted coefficients matrix using a bit number of simple arithmetic operations. In this regard, peers sustain very low computational complexities. As a result, the MATIN permits random network coding to be more efficient in P2P video streaming systems. The results obtained from simulation using OMNET++ show that it substantially outperforms the RNC which uses the Gauss-Jordan elimination method by providing better video quality on peers in terms of the four important performance metrics including video distortion, dependency distortion, End-to-End delay and Initial Startup delay.

## Introduction

Nowadays, live video multicasting has been of great interest among the users. More than 1.4 billion Internet users, near three billion gadgets, annual global IP traffic about 667 Exabyte in 2013 [1] are convincing reasons for this assertion. In this regard, the necessity of having efficient video multicasting technique is inevitable. Recently proposed methods such as IP multicasting [2] either probe to improve or change the extant routing algorithms and related parameters to QoS (Quality-of-Service) for providing higher performance. However, any change in the IP layer or the functions of routers involves high expense. Overlay networks [1], which can be established over the underlying network, support efficient multicasting without modifying network layer and routers. It seems necessary to be pointed out that most of the P2P systems [3] are implemented as an overlay on top of the underlying network. Based upon the topology, P2P systems are mainly categorized into Mesh- and Tree-based networks [4]. Single-Tree and Multi-Tree structures are two types of tree-based P2P networks. Tree-based networks are not resilient in peer churning, because the tree divides into two sub-trees when a node leaves the network which results in high cost in the reconstruction process and low video quality on peers due to many playback skips. Moreover, no leaf can participate in data dissemination which results in low network throughput [5]. Multi-Tree systems such as NICE [6] are introduced to cope with this problem. In a Multi-tree-based network, each peer is as an internal node in only one subtree and as a leaf node in others. However, other challenges of tree networks exist. Mesh-based networks are introduced to overcome mentioned challenges in tree-based P2P networks [7]. They are more robust in peer churning thanks to using pull-based exchange method [8] and redundant links among peers. Peers have also good opportunities to share their resources such as upload bandwidth for providing higher video quality in a mesh-based P2P network [9]. CoolStreaming [10] and SopCast [11] are two recently successful mesh-based P2P systems. It is possible to provide smooth video playback on peers in a P2P system if they encounter very few numbers of playback skips.

**Figure 1:**
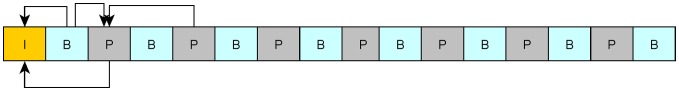
A GoP Consists of Sixteen Frames.

RNC has established this fact that it can be a suitable technique in multicasting video streams so that the probability of playback skip can considerably be decreased [12]. That is why P2P live video streaming using RNC is one of the most recently used systems for providing smooth video playback on peers. Actually, RNC considerably increases the error resiliency and the throughput of the network [13]. Better video quality can be achieved if peers exploit the benefits of using efficient video compression standard such as the H.264/MPEG (Moving Picture Experts Group) [14], [15]. In this regard, a live video source compresses video frames and arranges them in a GoP (Group-of-Pictures). Figure 1 depicts a GoP including 16 frames with one frames B between frames I and P (G16B1). Frame I (Intra-frame) is a reference frame for decoding all frames P and B, while each frame P (Predicted-frame) depends on previous frames I or P and each frame B (Bi-predictive-frame) can be decoded if both previous and next frames I and P have been successfully received and decoded. In a GoP, existing dependencies among its frames can result in many playback skips if, for example, a peer receives all frames P and B and frame I is not received yet. In conformity with the next sections, recent studies have shown that the integration of P2P networking and RNC in figure of a system can efficiently address mentioned challenges by increasing the network throughput and frame diversity [16]. However, high transmission overhead and computational complexity due to using RNC are remaining open issues in such a system. To address these issues, this study introduces the MATIN framework so that RNC can be more efficient in a P2P network.

The rest of this paper is organized as follows. Next sections explain random network coding, the problem statement, the MATIN framework and simulation results, respectively. Finally, the paper is concluded in the last section.

## Random Network Coding

**Figure 2:**
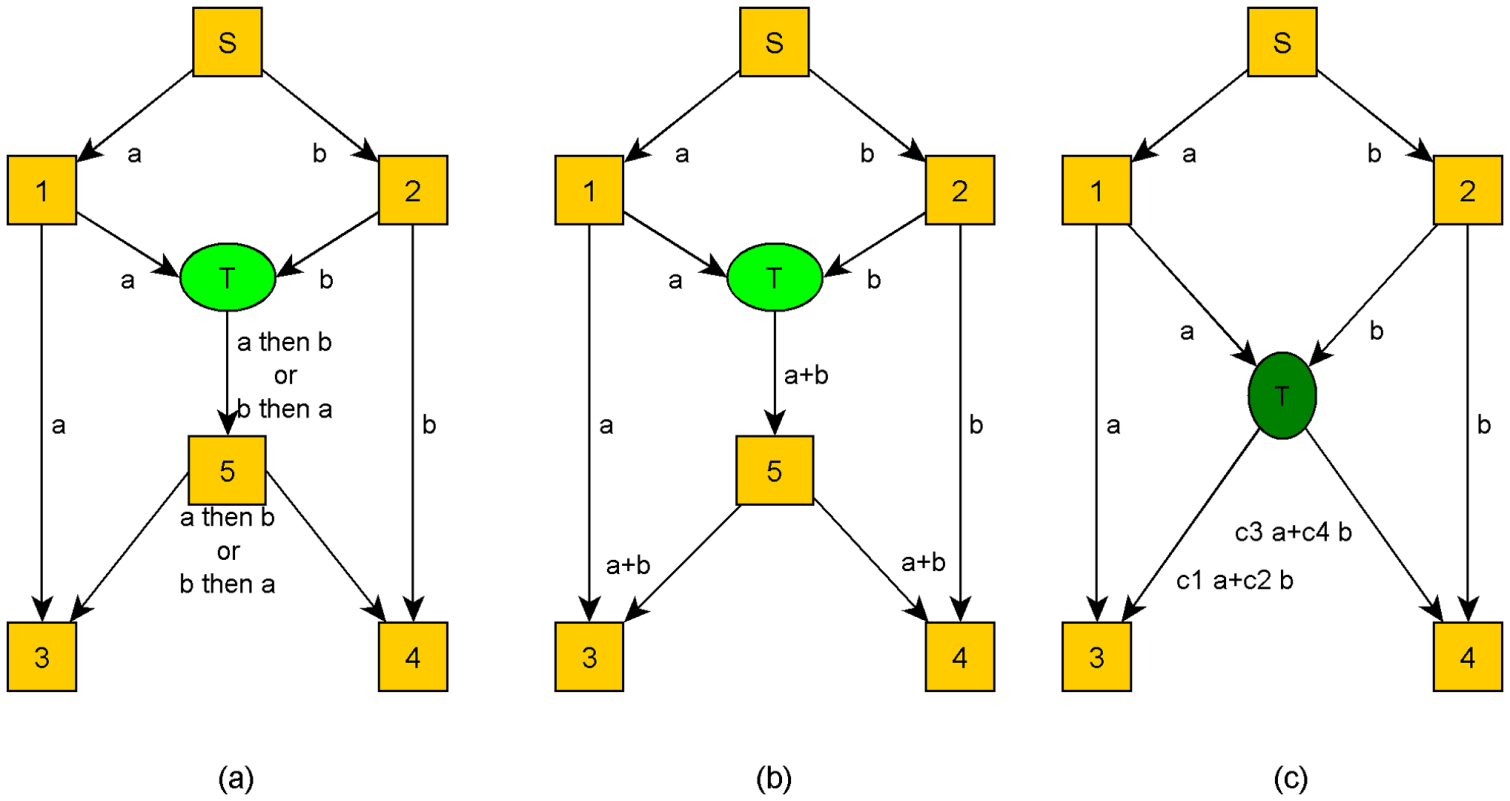
Packet forwarding without (a) and with network coding (b) and RNC (c).

In recent years, network coding promises an efficient solution for high quality video multicasting, especially in P2P systems [16]–[18]. Network coding was introduced by R. W. Yeung and Z. Zhang as an alternative to routing in 1999 [19]. This method considerably addresses the side effects of peer churning in a P2P system by increasing the packet diversity in the network. The simplest network coding method combines received packets using a simple XoR logical operation as it is depicted in Figure 2(b). In this figure, peer *T* performs one less transmission using XoR-based network coding 20] which results in higher network throughput. Moreover, peers 3 and 4 have their requested packets *b* and *a* in lower end-to-end delay, respectively. Although this method has shown that it can increase video quality in P2P systems 21,22], it is topology dependent and needs intelligent algorithms in encoding process for increasing the decoding probability among receivers as much as possible [22].

RNC, which is originally proposed by *Ho et al.* [23], not only addresses these problems, but also does not require any centralized knowledge about the topology of the network. In other words, it is completely topology independent. This is why many previous studies employed it in P2P video streaming systems [12], [16], [24]–[28]. Using RNC, the video stream can be divided into many fixed size segments, each of them is further divided into *n k*-byte blocks *B_i_ = [b_i1_,b_i2_,…,b_ik_],i = 1,2,...,n*. All blocks *B_i_* related to a segment can be arranged in a matrix, named *B_n×k_*. Then, the video source selects *n^2^* random values over finite field GF(2^m^) (Galois Field) 29,30] (*m* is an integral value) and arranges them in the coefficients matrix *C_n×n_*. Equations 1 and 2 show encoding and decoding processes in a sender and a receiver, respectively. Each peer attaches the coefficients vector *C_i_ = [c_i1_,c_i2_,…,c_in_],i = 1,2,...,n* to the encoded block *X_i_ = [x_i1_,x_i2_,…,x_ik_],i = 1,2,...,n* and sends them to the next hop as an encapsulated packet. A receiver can decode the encode segment if it can receive *n* linearly independent encoded blocks *X_i_*. All arithmetic operations are performed in GF(2^m^). For example, suppose that *g_1_*∈GF(2^m^), then, *g_1_*+*g_1_* = *0* and *g_1_*×*0* = *0*. In Figure 2(c), peer *T* selects four random coefficients *c1* to *c4* and generates two encoded packets before sending them to peers 3 and 4.
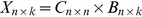
(1)

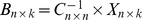
(2)


The decoding probability is an important parameter in selecting suitable values for *m*, because the number of innovative packets can be affected by the amount of this parameter. All previous studies used GF(2^8^) which is sufficient for data dissemination in a network with hundreds of links.

All in all, RNC improves network throughput and video frame localization. It also decreases playback skips, video distortion and delivery delay in the network. We refer interested researchers to the mentioned references for more information about the benefits of using network coding, especially RNC, in P2P streaming. Actually, the main goal of this study is not to prove the efficiency of RNC in P2P live video streaming. We aim to address existing challenges in RNC which are mentioned in previous studies and are categorized in the next section. Consequently, RNC can be more efficient in a P2P video streaming system using the MATIN framework.

## Problem Statement

What can be inferred from previous studies is that RNC is an efficient method for video multicasting over P2P networks and can be one of the most attractive research areas in the computer communication field in near future. However, according to these studies, RNC introduces new challenges to the system which are categorized as follows:

- As it was mentioned before, by using RNC, each peer attaches *n*-byte coefficients vector *C_i_* to each *k*-byte encoded block as its header before sending it to the next hop in the network. This results in high transmission overhead, especially when the values of *k* and *n* are small and large, respectively [12], [24]. This problem considerably degrades the video quality on peers, because they must assign a large portion of their upload bandwidth for transferring coefficients vectors *C_i_* as header to other peers. Obviously, the more number of video segments is transferred in each transmission, the better video quality can be provided on receivers. This can be achieved if the header size of an encoded blocks decreases using an efficient coefficients matrix generation method, because the peer can transfer next video blocks instead of coefficients vectors related to the previous video blocks.
- As soon as an encoded block receives, in the decoding step, peers use the Gauss-Jordan elimination method [31] for calculating the orthogonal matrix from the given coefficients matrix *C_n×n_* and checking any linear dependency among its vectors. If there is any linear dependency between two vectors, the decoder fails to decode the whole segment [12]. Moreover, the number of innovative packets will be decreased. These make two big problems. First, the decoder cannot send the decoded video segment to the buffer for extracting the video frames and playing by the media player. Therefore, the number of playback skips and the amount of the video distortion will be increased on this peer. Second, considering segment *S*, suppose that *i^th^* coefficients vector (*C_i_ ,i = 1,2,…,n*), which is related to the *i^th^* block (*Xi*), has linear dependency with another one (*C_j_*,*X_j_*) in this segment. The peer needs to wait for the same block number *i* but with different coefficients vector from another or the same sender. As it was mentioned earlier, this not only decreases the video quality on peer, but also wastes its computation processing power, because the peer needs to perform additional processing for decoding the new received encoded block. In summary, the Gauss-Jordan elimination method consumes high CPU (Central Processing Unit) time and imposes very high computational complexity on the system as they are mentioned in [20], [24], [32] and Table 1. This complexity causes many problems for small gadgets such as Smartphones which do not have enough processing powers [20], [33].
- The Gauss-Jordan elimination method needs to perform many arithmetic operations in decoding step. Therefore, the imposed computational complexity on the system considerably increases.

**Table 1:** Comparison of computational complexity in MATIN and RNC in use.

Operation	Current used approach in RNC	MATIN
Computational complexity for checking any linear dependency	Gauss–Jordan Elimination O(n^3^)	Absolutely Zero
Computational complexity for obtaining the inverted matrix C^−1^n×n	Gauss–Jordan Elimination O(n^3^) Traditional Method 1 M(O(n^2.697263^),O(n^3^))	2×n×D(8) 2 (Very Low)

1 Traditional method multiplies the inverted of the determinant value of the matrix (O(n^3^)) by the Adjoint of it (O(n^2.697263^)).

2 Computation complexity of dividing 1 byte by 1 byte.

As two solutions for overcoming these challenges, some previous studies suggested to decrease the packet batch size or increase the size of the finite field *m*. However, the first solution considerably degrades the performance of the network and video quality on peers [34], because more numbers of transmissions need to be performed. Actually, according to this fact that each packet is anchored with a header, this solution noticeably increases the network overhead. Using the second solution, although the decoding probability reaches nearby 1 using large values of *m* (e.g. *m* = 16), the computational complexity in encoding/decoding process and the required bandwidth for segment transmission sharply increases [20]. In addition, all peers need to check linear dependency in the coefficients matrix *C_n×n_* irrespective of its assigned values.

Therefore, the big question of this study puts forward as follows: Is there any efficient solution which not only addresses these problems, but also improves the performance of the RNC in a P2P live video streaming system? Next section will propose the MATIN to answer this question and efficiently address the mentioned challenges in this section. Moreover, there is no need to use parallel decoding algorithms which both imposes high energy cost on the system and needs considerable CPU resources in all peers [20].

## The Matin Framework

### A.1 Introduction to the Framework

**Figure 3:**
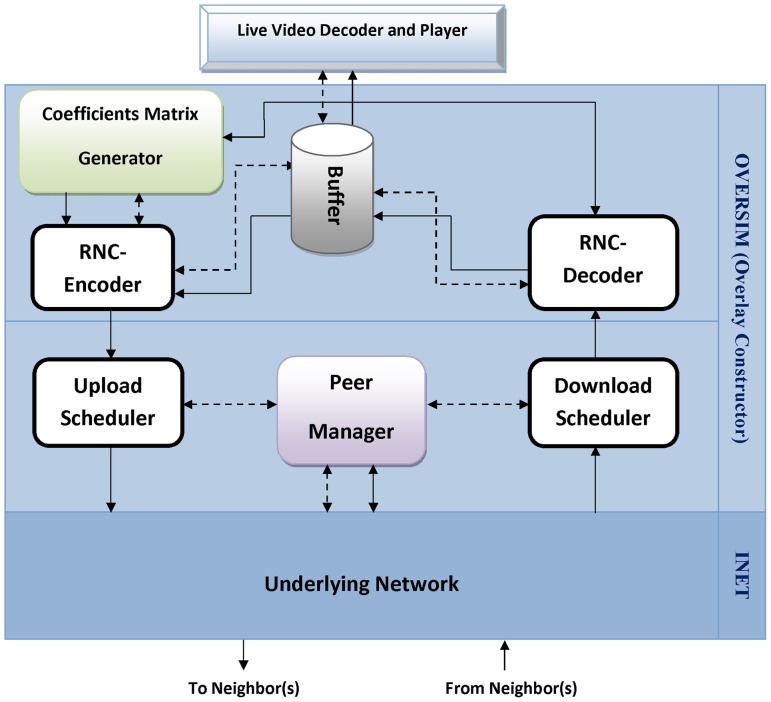
The MATIN Framework.

This section introduces MATIN, which includes a novel coefficients matrix generation method, in figure of a tailored framework so that it impressively addresses existing challenges in RNC. In summary, it:

- sharply decreases the imposed transmission overhead by RNC,
- completely removes the necessity of checking any linear dependency among the coefficients vectors. This increases the number of innovative packets in the network,
- considerably reduces the imposed decoding computational complexity,

As illustrated in Figure 3, the MATIN consists of some components. These components are as follows:

- *RNC-Encoder*: it receives original video segments from the buffer and applies RNC on them. Then, it encapsulates each coefficient entry *a_i_* and encoded block *X_i_* into a packet *P_i_* before sending them to the upload scheduler. Section A.2 explains this process in more details.
- *RNC-Decoder*: this component progressively decodes the received encoded blocks *X_i_* (*i = 1,2,…,n*) in order to regenerate the original video blocks *B_i_*. Section A.4 particularly discusses on this process.
- *Buffer*: all decoded video segments are stored in the buffer. These video segments generate the sequence of video frames for playing in the media player.
- *Peer Manager*: joining, leaving and other related managerial processes to the peer management are performed by this component.
- *Upload/Download Schedulers*: these components are responsible for transferring/receiving the encoded blocks to/from other neighbors in the system.
- *Coefficients Matrix Generator*: this component is the most important component of the MATIN which includes the contribution of this study. Actually, it efficiently generates the required coefficients matrix and its inversion as it is discussed in section A.2.2.

In this framework, solid and dashed lines refer to the data and the control messages, respectively.

### A.2 Encoding Process

#### A.2.1 Introduction to Encoding

As soon as the RNC-Encoder intends to encode segment *B_n×k_*, it performs the following steps respectively:

- It first divides the segment into *n k*-byte blocks.
- Then, this component sends a request message (REQ_MSG) containing the number of blocks (*n*) to the coefficients matrix generator component.
- After receiving the coefficients matrix *C_n×n,_* the RNC-Encoder generates *n* encoded blocks *X_i_* using Equation 1. All arithmetic operations are performed over GF (2^m^).
- Finally, it attaches coefficient entry *a_i_* *εA_1_×_n_* to the encoded blocks *X_i_* in figure of packet *P_i_* before sending it to the upload scheduler for transferring to receivers.

This process can be performed for many times until the peer either leaves the network or the video streaming finishes. Algorithms 1 and 2 show the encoding process when the most recently used method, the Gauss-Jordan elimination, and the MATIN framework are employed in the system, respectively.

*Algorithm 1.* Generating encoded blocks in the current RNC RNC in used

1. To generate the encoded blocks *X_i_, i = 1,2,...,n*
  - 1.1. Select *n^2^* random values over field GF(2^m^)
  - 1.2. Generate *C_n×n_* based on *n^2^* values
  - 1.3. Generate all encoded blocks *X_i_* using the Equation 1
2. For all *Xi*, *i = 1,2,...,n*
  - 2.1. For all *C_i_  = [c_i1_,c_i2_,…,c_in_]* ε*C_n×n_,i = 1,2,...,n*
    - 2.1.1. Encapsulate *C_i_* and *X_i_* into Packet *P_i_*
    - 2.1.2. Send *P_i_* to the next hop // *next peer(s) in the network*

*Algorithm 2.* Generating encoded blocks using MATIN framework

1. To generate the encoded blocks *X_i_, i = 1,2,...,n*
  - 1.1. Select *n* random values over field GF(2^m^)-{zero} and arrange them in *A_1×n_*
  - 1.2. Generate *C_n×n_* from the given matrix *A_1×n_*
  - 1.3. Generate all encoded blocks *X_i_* using the Equation 1 in RNC-Encoder Component

2. For all *X_i_, i = 1,2,...,n*
  - 2.1. For all *a_i_*ε*A_1×n_, i = 1,2,...,n*
    - 2.1.1. Encapsulate *a_i_* and *X_i_* into Packet *P_i_*
    - 2.1.2. Send *P_i_* to the next hop // *next peer(s) in the network.*

#### A.2.2 Coefficients Matrix Generation and Inversion

By receiving a request from the RNC-Encoder, this component first selects *n* random values over field GF(2^m^)-{zero} and arranges them in matrix *A_1×n_ = [a_1_,a_2_,…,a_n_]*. Recall that, current RNC in use needs *n^2^* random values for generating the matrix *C_n×n_*. Based upon *A_1_*_×*n*_, the diagonal matrix *A_n×n_* can be generated as follows. Definitely, the determinant value of the matrix *A_n×n_* is not equal to zero (det(*A_n×n_*)≠0); because all entries *a_i_*≠0. Therefore, the matrix *A_n×n_* is undoubtedly invertible. MATIN does not consider *A_n×n_* as the final coefficients matrix for sending to the RNC-Encoder, because having many zero values in the coefficients matrix can decrease the decoding probability. In order to resolve this problem, it is possible to add some multiplication of one row/column of *A_n×n_* to another row/column. Certainly, the value of the det(*A_n×n_*) will not change [35].
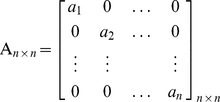



In this regard, for generating the coefficients matrix *C_n×n_*, this component follows the following rules:

*Rule i:* It replaces *j^th^* column of *A_n×n_* by column *j* plus column *j-1*, for *j = 2,3,…,n,* respectively.

*Rule ii:* Then, it replaces *j^th^* column of *A_n×n_* by multiplying 3 by column *j* plus column *n*, for *j = 1,2,..,n-1,* respectively.

Now, the coefficients matrix *C_n×n_* can be sent to RNC-Encoder as follows (recall that, all arithmetic operations are performed in GF (2^m^)).
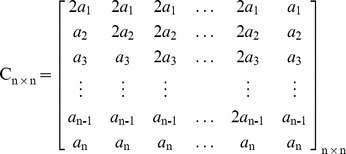



After applying the second rule, det(*C_n×n_)* = det(*A_n×n_*)×3^n−1^≠0. Thus, matrix *C_n×n_*, similar to the *A_n×n_*, will be undoubtedly invertible and no linear dependency exists among its vectors. It is necessary to be pointed out that a received packet in a peer can be innovative if it does not have any linear dependency with the current received packets in its buffer [36]. Therefore, the number of innovative packets can be increased and peers can have their required video packets in less number of transmissions and lower delay. Actually, there is no need to send another encoded packet due to existing linear dependency between the current received packet and the packets in the buffer. Obviously, any change in *A_1×n_* will alter *C_n×n_* uniquely, because the *n^th^* column of *C_n×n_* is the transposed of *A_1×n_*. It is necessary to emphasize that each peer directly obtains *C_n×n_* from the given *A_1_*_×*n*_. Consequently, there is no additional computational complexity in coefficients matrix generation process.

Based upon the matrix *C_n×n_*, the inverted coefficients matrix *C^-1^_n×n_* can be shown as follows. As can be seen in this matrix, just two entries are needed to be calculated for obtaining each row, because other entries are equal to zero. In this regard, contrary to the Gauss-Jordan elimination method, the imposed computational complexity due to obtaining the inverted coefficients matrix and decoding the encoded blocks sharply decreases. This allows receivers, especially Smartphones, to assign very low CPU processing power to decoders. As it was mentioned before, high computational complexity in decoding is an important concern for those users who use small gadgets in a P2P system which uses RNC for multicasting.
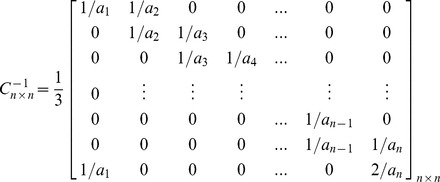



In order to prove that *C^−1^_n×n_* is the unique inverted matrix of *C_n×n_*, it is sufficient to show that *I_n×n_ = C_n×n_×C^−1^_n×n_ =  C^−1^_n×n_×C_n×n_* [35] as follows: 
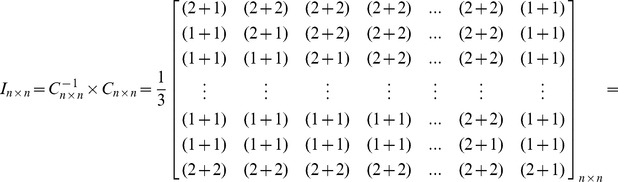


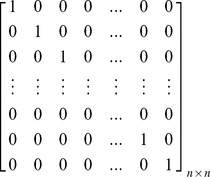



In the same manner, it is possible to prove that *I_n×n_ = C_n×n_×C^−1^_n×n_*. Therefore, *C^−1^_n×n_* is the unique inverted matrix of *C_n×n_*.

### A.3 Transmission Overhead

Transmission overhead in the MATIN is very low, because the encoder attaches each entry of the matrix *A_1×n_* instead of each vector *C_i_*ε*C_n×n_* to the encoded block *X_i_*. For instance, assume that each entry of all matrices is one byte in GF(2^8^). Therefore, the data transmission overhead in Algorithms 1 and 2 can be written as Equations 3 and 4, respectively (*n* and *k* are in byte).
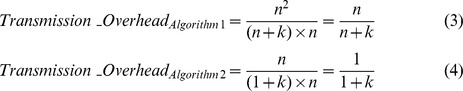



According to *Mirshokraie et. al*. [37], network coding complexity and transmission overhead sharply heightens when the number of blocks (*n*) increases. However, as depicted in Equation 4, transmission overhead of the MATIN is completely independent of the number of blocks. It means that the MATIN lets us ignore both the imposed computational complexity and the transmission overhead due to employing and sending the coefficients matrix *C_n×n_*, respectively. As a result, better video quality can be provided on peers.

### A.4 Decoding process

Similar to the Gauss-Jordan elimination method, the decoding process can be performed progressively. The MATIN progressively starts to generate the matrix *C^-1^_n×n_* using small numbers of arithmetic operations when the first entry *a_1_* is received. Suppose *C^−1^_n×n_* is written as follows and node P1 is receiving segment *X_n×k_*. Obviously, each encoded block *X_i_* is anchored by a coefficient entry *a_i_* and the value of *i* is determined in the header. The decoding process in P1 can be performed in the following order:



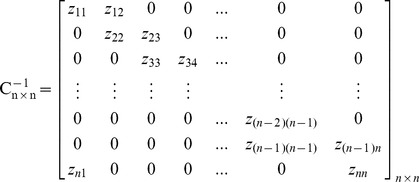


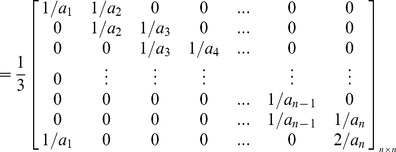

i. It first sets all values *b_ir_*ε*B_n×k_* (*i = 1,2,…,n, r = 1,2,…,k*) to zero before starting the decoding process.
ii. As soon as a coefficient entry *a_i_* is received, P1 generates two related entries *z_ij_*ε*C^−1^_n×n_ (i,j = 1,2,...,n)*. For example, suppose that P1 receives packet *P_1_* = (*a_1_,X_1_*). In this case, P1 can easily generate *z_11_* and *z_n1_* from the given *a_1_* as depicted in the first row of Table 2 (Iteration 1).
iii. Finally, P1 sends the calculated *z_ij_* to the Function 1 for progressively obtaining entries *b_ir_*ε*B_n×k_.* This function will not be called for zero entries and *z_ij_* = 0 in matrix *C^−1^_n×n_*, because the value of *b_ir_* will not be changed in this case. This considerably decreases the imposed computational complexity in decoding.

**Table 2:** Progressively Decoding in MATIN Using Function 1.

Iteration	Received Packet	Received Coefficient Entry	Obtained *z_ij_*Based upon the given *a_i_*	Calculating *b_ir_* Progressively
1	*p_1 = _(a_1_,X_1_)*	*a_1_*	*z_n1_ = z_11_ = (1/3)×(1/a_1_)*	*b_11_ = 0+(z_11_×x_11_)*
				*b_12_ = 0+(z_11_×x_12_)*
				…
				*b_n1_ = 0+(z_n1_×x_11_)*
				*b_n2_ = 0+(z_n1_×x_12_)*
				…
2	*p_2 = _(a_2_,X_2_)*	*a_2_*	*z_12_ = z_22_ = (1/3)×(1/a_2_)*	*b_11_ = b_11_+(z_12_×x_21_)*
				*b_12_ = b_12_+(z_12_×x_22_)*
				…
				*b_21_ = 0+(z_22_×x_21_)*
				*b_22_ = 0+(z_22_×x_22_)*
⋮	⋮	⋮	⋮	⋮
n	*p_n = _(a_n_,X_n_)*	*a_n_*	*z_nn_ = (1/3)×(2/a_n_) z_(n-1)n_ = (1/3)×(1/a_n_)*	*b_(n-1)k_ = 0+(z_(n-1)n_×x_nk_)*
				…
				*b_nk_ = b_nk_+(z_nn_×x_nk_)*

P1 will repeat steps ii and iii until all entries *b_ir_*ε*B_n×k_* are calculated. This process can be performed for other received encoded segments. In contrary to *C_n×n_*, having many zero values in the matrix *C^-1^_n×n_* leads to very low computational complexity in decoding. This is another reason for this fact that the MATIN sharply decreases the computational complexity of RNC.

*Function 1.* Progressively Decoding Process in MATIN

Function MATIN_Decoder (*z_ij_*, *i*, *j*)

For *r* = 1 to *k* Do

*b_ir_* = *b_ir_*+(*z_ij_*×*x_jr_*)

EndFor

If *i* = *j* = 1Then *// because z_n1_ = z_11_*

For *r* = 1 to *k* Do

*b_nr_* = *b_nr_*+(*z_ij_*×*x_jr_*)

EndFor

EndIf

End

In order to illuminate the operation of Function 1, suppose that node P1 receives encoded bock *X_i_* in iteration *i*. This Function can be called two times in each iteration (e.g. one call for *z_11_* and one call for *z_n1_* in iteration 1). Table 2 shows the decoding process. In this table, *b_11_* can be obtained at the end of the second iteration, because *z_1j_ = 0 (j = 3,4,5,...,n)* will not be sent to Function 1. This is the same for *b_12_*_._ In this regard, *b_21_* and *b_22_* can be obtained at the end of the iteration 3 and so on. Suppose that *X_i_* is not received or received with error. In this case, the MATIN, just like as the Gauss-Jordan elimination method, can use the received encoded blocks *X^″^_i_* from another node instead of *X_i_,* because both of them are generated based upon the same matrix *B_n×k_* but with different coefficients matrices *C^″^_n×n_* and *C_n×n_*, respectively.

Finally, it is necessary to be pointed out that each peer downloads a small source code in size for just one time when it joins the network. This source code can be used by peers in order to generate the same coefficients matrix *C_n×n_* based upon the same given matrix *A_1×n_*.

### A.5 Complexity Analysis

**Figure 4:**
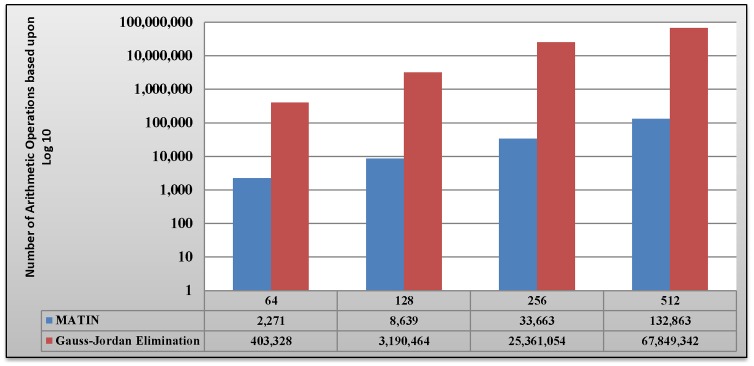
A Comparison between required arithmetic operations in MATIN and the Gauss-Jordan Elimination.

As it was mentioned before, the MATIN imposes very low computational complexity on the system. Figure 4 compares the required number of arithmetic operations for n =  [64, 128, 256, 512] using the MATIN (in encoding and decoding) and the Gauss-Jordan elimination (in just decoding). As emphasized in [33], RNC imposes high computation complexity on gadgets (e.g. Smartphones) so that they need to assign considerable CPU time in decoding process. On the other hand, using the MATIN, this concern can be completely ignored, because it imposes very low computation complexity on peers and needs a bit CPU time for decoding the received encoded blocks. Therefore, RNC can be more useful.

In order to provide a more comprehensive analysis over MATIN, suppose that there are *n k*-byte blocks for encoding over GF(2*^m^*). Based upon sections A.2 to A.4, it is possible to calculate the complexity of MATIN as follows:

• Throughput: There is no linear dependency in the generated encoded packets using MATIN. Therefore, all received packets can be as an innovative packet. This removes the necessity of re-transmission due to linear dependency between two received encoded packets. Moreover, because of low transmission overhead, bandwidth utilization considerably increases meaning that MATIN provides very high performance (optimal) in terms of the throughput.
• Algorithm Complexity: Suppose that generating one random coefficient entry imposes constant complexity of *G*. Moreover, addition/multiplication operation imposes constant complexity of *A.* Generating the matrix *A_1×n_* and applying rules i and ii impose a complexity of *O(nG*)+*O(n^2^A)*. Therefore, the complexity of the algorithm will be *O(n^2^A)*.
• Encoding Complexity: MATIN generates the coefficients matrix *C_n×n_* based upon *n* random values in GF(2*^m^*). Then, the encoder multiplies *C_n×n_* by *B_n×k_* which leads to complexity of *O(n^2^k)*.
• Decoding Complexity: According to section 4.4, MATIN imposes very low computational complexity on the system in decoding. In fact, just 2×*n* entries are needed to be calculated for obtaining *C^−1^_n×n_* (2 entries in each row). This just leads to decoding complexity of *O(nk)* in the system.

Packet Overhead in bit: According to section 4.3, RNC-Encoder attaches one instead of *n* coefficient entries to each encoded block. Therefore, the imposed packet overhead by it is equal to *m* bits.
Packet Feedback: Similar to RNC, MATIN needs no feedback.

Table 3 summarizes the complexity of MATIN. According to *Qureshi et. al.(2012)* [20], MATIN outperforms existing coding schemes in terms of the considered parameters. In their study, parameters *q*, *M* and *B* are defined the same as *m*, *n* and *k* in our study, respectively.

**Table 3:** The MATIN's Complexity based Upon Six Important Parameters.

Parameter	MATIN
Throughput	Very High (Optimal)
Algorithm Complexity	O(*n^2^A*)
Encoding Complexity	O(*n^2^k*)
Decoding Complexity	O(*nk*)
Packet Overhead (bits)	*m*
Packet Feedback	Not Required

## Simulation

### B.1 Simulation Parameters

For better understanding and evaluating the performance of the MATIN in P2P live video streaming, this study carried out a simulation using the INET and the OVERSIM frameworks in OMNET++ [38]. Then, it compares the MATIN with RNC-GJE (the RNC which uses the most recently used method, the Gauss-Jordan elimination, in decoding). The OMNET++ is a discrete-event-based simulator which includes many C++ libraries and frameworks such as the OVERSIM for overlay construction and the INET for underlying layer operations. Table 4 depicts different considered parameters and their values in this simulation.

**Table 4:** Considered Parameters and Their Values in the Simulation.

Parameter	Value(s)	Parameter	Value(s)
Video Stream Type	Variable Bit Rate (VBR)	Number of Neighbors	Uniform(4,6)
MTU	1500 Bytes	Live Video Stream Length	600 Second
Network Size	150,300	Peer Distribution Model	Random
Confidence Interval	95 Percent	Segment Size	One Second
Initial Buffer Time	8 Seconds	Block Size (*k*)	128,512,1024 Bytes
Underlying Network	INET Framework	Overlay Constructor	OVERSIM Framework
Peer Churning Scenarios	Scenario 1: No Churn (All peers remain in the network up to the end of the simulation)		
	Scenario 2: Uniform(1,3) and Peer Lifetime Mean = 400 Seconds (Peers join the network every T second (T = Random(1,3))		
	Scenario 3: Uniform(0.5,1) and Peer Lifetime Mean = 300 Seconds (Peers join the network every T second (T = Random(0.5,1))		

The video source disseminates Star War IV, a single-layer MPEG-4 video trace file available from [39]. Table 5 shows the characteristics of the video stream. The video stream is divided into many segments based upon the considered segment size in Table 4, each of them is further divided into *n k*-byte blocks (*k* = [128,512,1024]). The simulation ran for five times based upon the mentioned conditions and parameters in Tables 4 and 5 and the results are depicted in figures with 95% confidence interval (CI). According to Table 4, the performance of MATIN is evaluated using different churning scenarios. This makes the evaluation process more comprehensive, because the behavior of the network can be more similar to real-world systems. In this simulation, all arithmetic operations are performed in GF(2^8^).

**Table 5:** Live Video Stream Characteristics.

Video File	Frame Per Second	Layer	GoP Structure	Quantizer Parameter	Mean Frame Size	Mean Frame PSNR	Mean Frame Bit Rate
STAR WAR IV	30	Single	G16B1	8	631.64 B	37.35 dB	151595.34 bps

#### B.2 Simulation Results and Discussion

In order to measure the performance provided by the MATIN and the RNC-GJE, this study considers four important performance metrics as follows:

• *Video Distortion*: The capacity of frames not playback divided by the total capacities of all video frames of the stream.
• *End-to-End Delay (EED)*: The time elapsed between transmitting a video frame from the video source and playing it in a peer.
• *Initial Startup Delay (ISD)*: The time elapsed between starting to buffer the video frames after joining the network and playing the first buffered video frame. In other words, ISD shows how much time it took in a peer to finish the initial buffer time after joining the network.
• *Dependency Distortion*: According to the existing dependencies among video frames, it is possible that a video frame arrives, however, the decoder cannot decode it due to this fact that it is dependent on a frame which has not received yet. For example, peer P receives frame B3, while frames P1 and P2 are not received. Thus, it is impossible to successfully decode frame B3 (by video decoder not RNC-Decoder) and playback it. This metric shows the capacities of these frames divided by the total capacity of all video frames in percentage.

This section is divided into three subsections; each of them evaluates the obtained results related to one of the considered churning scenarios. In all scenarios, we use aggregation method so that each packet can contain more than one encoded block. By employing aggregation approach, suppose that *P_G_* and *P_M_* indicate the number of encoded blocks in a packet using the RNC-GJE and MATIN, respectively (*MTU* shows the maximum transfer units in byte). In this regard, according to Equations 5 and 6, *G_G_* and *G_M_* depict the number of generated packets for transmitting a segment to a neighbor using the RNC-GJE and MATIN, respectively.
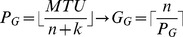
(5)

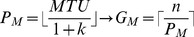
(6)


Then, for 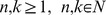:
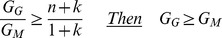



This clearly indicates that RNC-GJE imposes higher traffic on the network which can result in higher video distortion, because using same block and segment size, the capacity of all generated packets by the MATIN is lesser than that of the RNC-GJE.

#### B.2.1 Scenario 1: Results and Discussion

**Figure 5:**
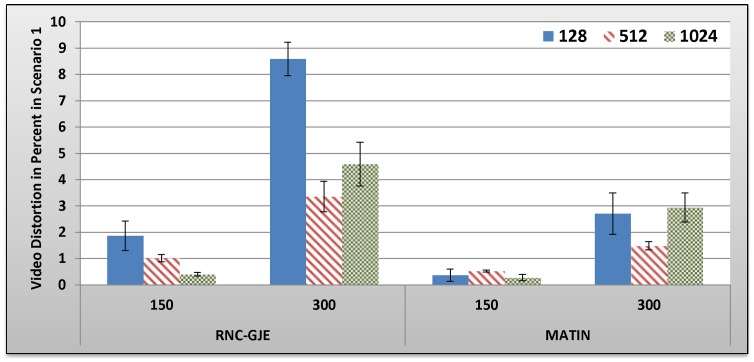
Experienced Video Distortion in Percent by 150 and 300 Peers in Scenario 1 with 95% CI for 128, 512 and 1024 Bytes Block Sizes.

**Figure 6:**
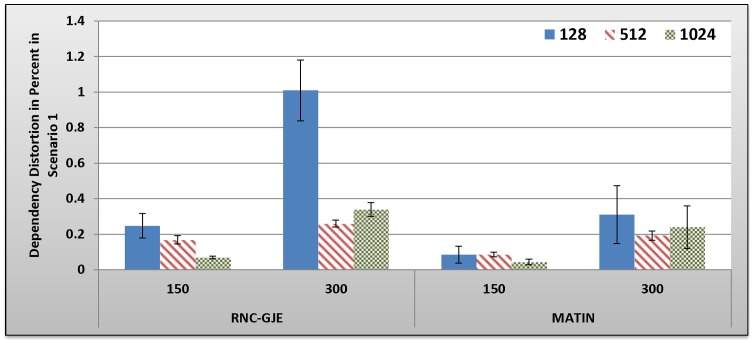
Experienced Dependency Distortion in Percent by 150 and 300 Peers in Scenario 1 with 95% CI for 128, 512 and 1024 Bytes Block Sizes.

**Figure 7:**
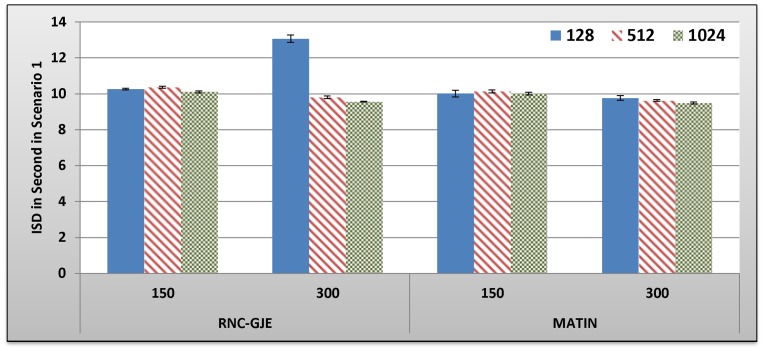
Experienced ISD in Second by 150 and 300 Peers in Scenario 1 with 95% CI for 128, 512 and 1024 Bytes Block Sizes.

**Figure 8:**
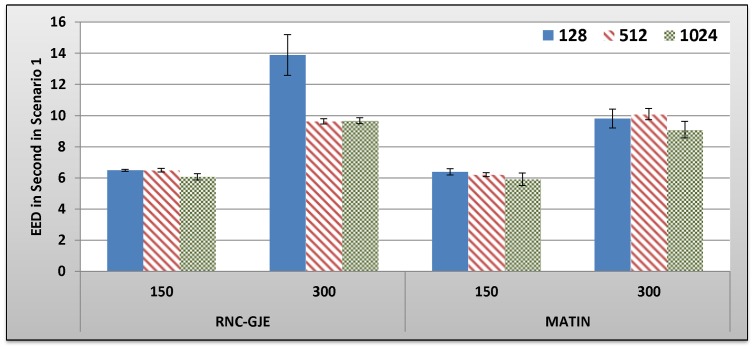
Experienced EED in Second by 150 and 300 Peers in Scenario 1 with 95% CI for 128, 512 and 1024 Bytes Block Sizes.

Figures 5, 6, 7, 8 show the amount of video distortion, dependency distortion, ISD and EED, respectively, with 95% confidence interval for different number of peers and block sizes ([128,512,1024]) when there is no churn in the network. In fact, all peers join the network in the beginning of the simulation and remain up to the end of the simulation.

In an overall view, considering 150 or 300 peers, what can be inferred from these figures is that the MATIN provides higher video quality than that of the RNC-GJE with slightly lower end-to-end and initial startup delays. According to Figures 5 and 6, the reason for high video distortion for block size 128-byte is that the video segment is divided into many blocks (the value of *n* is large). In this case, the RNC-GJE method needs to attach n bytes as header to each encoded block before encapsulating it in the packet. This increases the transmission overhead which causes high video distortion. The MATIN efficiently overcomes this problem by attaching just one instead of n bytes to each encoded blocks. For instance, suppose that a segment is divided into *n* = 10 *k* = 128-byte blocks. The imposed transmission overhead using the RNC-GJE and MATIN will be 4.4 and 0.78 percent, respectively. The difference between the imposed transmission overheads by these methods can be sharply increased when *n* increases.

Network size can influence the network performance. According to Figure 5, MATIN introduces less amount of video distortion to peers even if the network size increases from 150 to 300 peers. Using the RNC-GJE method, video distortion considerably increases when there are 300 peers in the network. This means that the MATIN permits the network to be more scalable. Recall that each encoded block *X*i is anchored by a coefficients vector *C_i_* using RNC-GJE method. In this regard, the amounts of Transmitted Data (TD) and the imposed transmission overhead reduce when the amount of *k* increases. This is why the imposed video distortions on peers slightly decrease for larger values of *k*. Using the MATIN framework, on the other hand, the amount of video distortion does not sharply increase even if the amount of *K* increases. This is more visible for 128-byte block size. This means that the MATIN can provide better video quality than that of the RNC-GJE even if the network size and block size increases and decreases, respectively. In order to substantiate this claim, consider 128-byte block size. Using MATIN, the introduced amount of video distortion only increases 0.83 in case of having 300 peers in comparison with the RNC-GJE when there are 150 peers in the network. Moreover, MATIN improves the video distortion about 508 percent in comparison with the RNC-GJE when there are 150 peers in the network and block size is 128 bytes. For the same network size (150 peers), 196 and 150 percent improvement are achieved when block size is equal to 512 and 1024 bytes, respectively. This is approximately the same for 300 peers.

**Figure 9:**
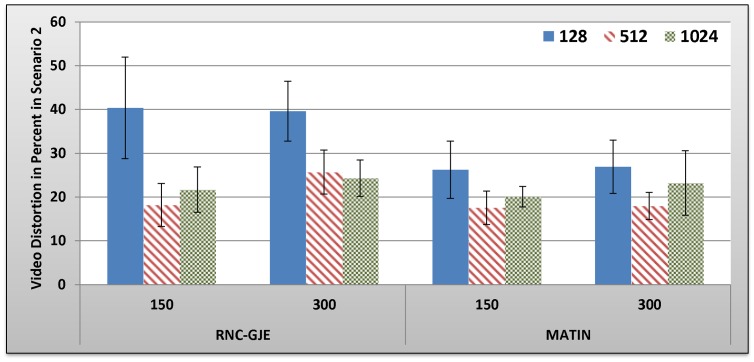
Experienced Video Distortion in Percent by 150 and 300 Peers in Scenario 2 with 95% CI for 128, 512 and 1024 Bytes Block Sizes.

Considering the RNC-GJE method and segment size *S*, the large number of generated packets (G_G_) due to using blocks size 128 bytes leads to high video distortion, especially when the network size increases. The reason is that the amounts of traffic and end-to-end delay considerably increase. However, using block size 512 bytes results in better video quality than that of block size 1024 bytes when there are 300 peers in the network. The main reason is that the amount of P_G_ increases while the amount of TD decreases. This leads to higher frame diversity in the network, because each packet includes more number of video blocks *X_i_*. Although the amount of P_G_ increases more when the block size is 128 bytes, the imposed transmission overhead and the large amount of TD outperform the advantages of having large amount of P_G_ so that the video distortion increases. The obtained results in Figures 5, 6, 7, 8, 9 truly confirm the propounded hypothesis in which the best block size can be 512 bytes [37].

Now, consider the same segment size *S* but the MATIN is used in the network. The amounts of TD and transmission overhead on the system remain low and almost constant even if the amount of *k* increases. This is why approximately the same amounts of video distortion are experienced by 150 peers. In case of large network size (e.g. 300 peers), the amount of the imposed transmission overhead plays more important role than that of other parameters (TD, P_M_ and G_M_). In fact, low frame diversity due to small number of P_M_ causes higher video distortion using block size 1024 bytes in comparison with the block size 512 bytes.

Dependency distortion is another important metric. This metric evaluates the performance of a method/framework in video streaming in a detailed view. As can be seen in Figure 6, the MATIN introduces less amount of dependency distortion to the system which results in higher bandwidth utilization. Actually, the bandwidth can be wasted if a video frame arrives at the destination peer, but the video decoder cannot decode it due to existing dependency between the frame and its reference frame. As depicted in Table 6 in section B.2.3, the MATIN provides such a high performance while it introduces a bit less amounts of end-to-end and initial startup delays to peers, especially for block size 128 bytes when there are 300 peers in the network. As a result, the MATIN considerably outperforms the RNC-GJE in stable networks when no peer churning happens. Next sections evaluate the performances of MATIN in churning scenarios where peers join and leave the network repeatedly.

**Table 6:** Comparison between MATIN and RNC-GJE Numerically.

Video Distortion in Percent												
Peers →	150						300					
Block Size →	128-Byte		512-Byte		1024-Byte		128-Byte		512-Byte		1024-Byte	
Method→	RNC-GJE	MATIN	RNC-GJE	MATIN	RNC-GJE	MATIN	RNC-GJE	MATIN	RNC-GJE	MATIN	RNC-GJE	MATIN
Scenario↓												
Scenario1	*1.868*	*0.367*	*1.017*	*0.525*	*0.405*	*0.279*	*8.588*	*2.708*	*3.354*	*1.488*	*4.595*	*2.942*
Scenario2	*40.35*	*26.22*	*181.6*	*17.52*	*21.67*	*20.04*	*35.59*	*26.9*	*25.72*	*17.96*	*24.29*	*23.23*
Scenario3	*28.65*	*22.24*	*26.04*	*20.39*	*23.99*	*19.27*	*29.39*	*18.96*	*18.78*	*18.54*	*23.78*	*18.02*
**Dependency Distortion in Percent**												
Scenario1	*0.246*	*0.084*	*0.168*	*0.085*	*0.068*	*0.043*	*1.009*	*0.310*	*0.259*	*0.192*	*0.338*	*0.239*
Scenario2	*4.305*	*1.022*	*1.478*	*0.985*	*1.097*	*1.396*	*3.138*	*1.450*	*1.457*	*0.710*	*0.977*	*0.864*
Scenario3	*3.099*	*2.238*	*0.632*	*0.976*	*1.278*	*1.223*	*2.837*	*0.556*	*0.954*	*0.918*	*0.626*	*0.425*
**Initial Startup Delay (ISD) in Second**												
Scenario1	*10.26*	*10.01*	*10.36*	*10.13*	*10.10*	*10.09*	*13.06*	*9.76*	*9.81*	*9.62*	*9.57*	*9.48*
Scenario2	*12.39*	*11.86*	*11.03*	*10.20*	*10.63*	*10.07*	*12.32*	*11.17*	*12.41*	*10.56*	*12.58*	*11.38*
Scenario3	*11.70*	*11.11*	*11.75*	*11.46*	*11.60*	*10.83*	*12.14*	*10.45*	*11.04*	*10.77*	*12.26*	*10.49*
**End-to-End Delay (EED) in Second**												
Scenario1	*6.49*	*6.38*	*6.49*	*6.21*	*6.07*	*5.90*	*13.89*	*9.81*	*9.63*	*10.09*	*9.67*	*9.10*
Scenario2	*17.62*	*13.64*	*10.37*	*10.61*	*11.74*	*11.09*	*16.66*	*12.86*	*14.76*	*8.87*	*14.28*	*11.16*
Scenario3	*10.76*	*10.06*	*9.45*	*10.64*	*11.99*	*11.41*	*9.50*	*10.54*	*10.70*	*10.66*	*10.55*	*9.75*

#### B.2.2 Scenario 2: Results and Discussion

**Figure 10:**
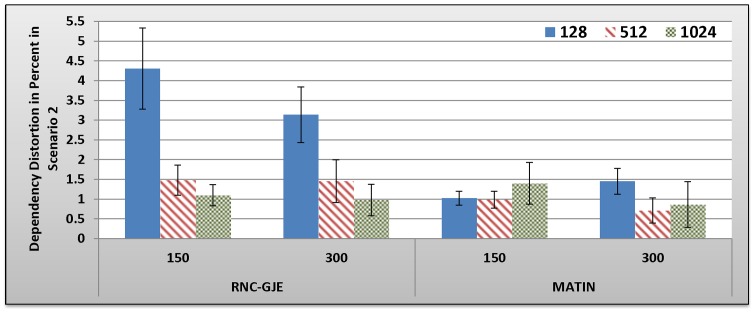
Experienced Dependency Distortion in Percent by 150 and 300 Peers in Scenario 2 with 95% CI for 128, 512 and 1024 Bytes Block Sizes.

**Figure 11:**
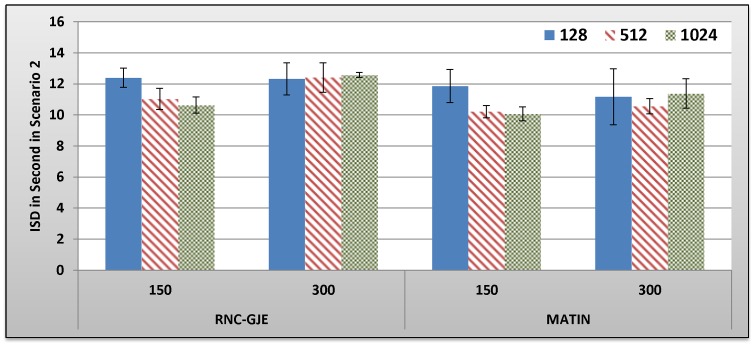
Experienced ISD in Second by 150 and 300 Peers in Scenario 2 with 95% CI for 128, 512 and 1024 Bytes Block Sizes.

**Figure 12:**
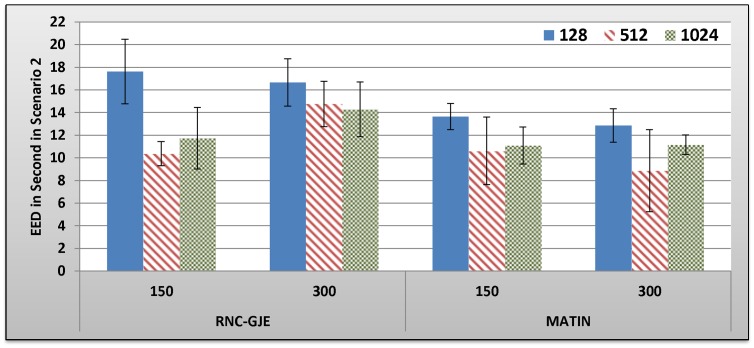
Experienced EED in Second by 150 and 300 Peers in Scenario 2 with 95% CI for 128, 512 and 1024 Bytes Block Sizes.

Figures 9, 10, 11, 12 show the amount of video distortion, dependency distortion, ISD and EED, respectively, with 95% confidence interval for different number of peers and block sizes ([128,512,1024]) when the second scenario is considered for peer churning. In this scenario, one peer joins the network every *T* seconds so that the first peer joins at the beginning of the simulation. The amount of *T* can be selected randomly between 1 and 3 seconds using normal distribution method. In addition, the averaged lifetime duration of peers in the network is 400 seconds. As can be seen in Figures 9, 10, 11, 12, the MATIN noticeably outperforms the RNC-GJE in terms of the video and dependency distortions. It also introduces lesser amounts of EED and ISD to the network. Obviously, churning causes higher video distortion in comparison with the first scenario which no churn happened in the network. The effects of churn are more visible when the block size is equal to 128 bytes. An interesting result in the second scenario is that, contrary to the first scenario, the perceived video qualities on peers remain almost the same when the network size increases from 150 to 300 peers using the MATIN framework.

This behavior is somehow the same for the RNC-GJE in block sizes 128 and 1024 bytes. However, the MATIN provides better video quality than that of the RNC-GJE by introducing lesser amount of video distortion on both 150 and 300 peers. Moreover, according to Figure 10, it considerably decreases the effects of frame dependency in decoding, even if peer churning exists in the network. Again, the MATIN introduces lesser amounts of EED and ISD to peers, even if the network size increases. This shows that the MATIN increases the network resiliency in dynamic networks where churning happens.

#### B.2.3 Scenario 3: Results and Discussion

**Figure 13:**
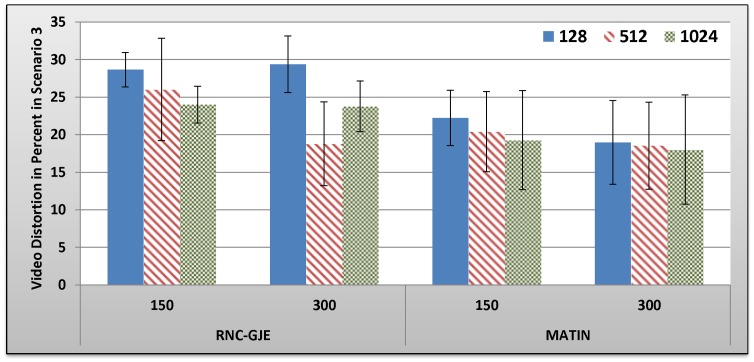
Experienced Video Distortion in Percent by 150 and 300 Peers in Scenario 3 with 95% CI for 128, 512 and 1024 Bytes Block Sizes.

**Figure 14:**
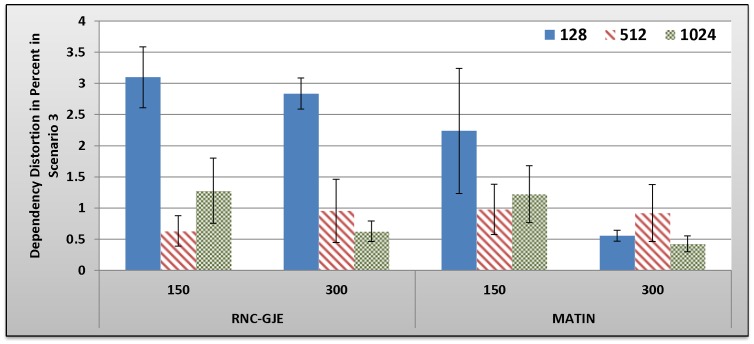
Experienced Dependency Distortion in Percent by 150 and 300 Peers in Scenario 3 with 95% CI for 128, 512 and 1024 Bytes Block Sizes.

**Figure 15:**
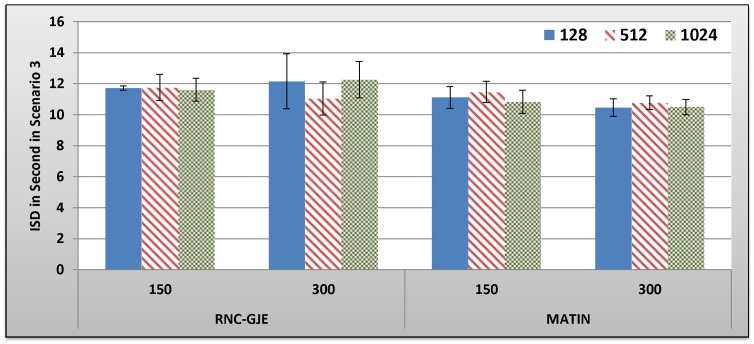
Experienced ISD in Second by 150 and 300 Peers in Scenario 3 with 95% CI for 128, 512 and 1024 Bytes Block Sizes.

**Figure 16:**
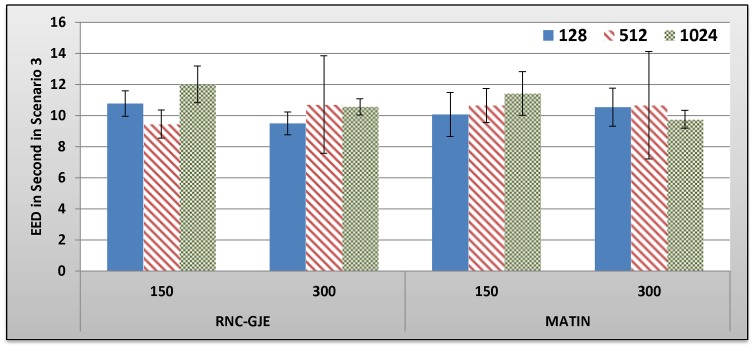
Experienced EED in Second by 150 and 300 Peers in Scenario 3 with 95% CI for 128, 512 and 1024 Bytes Block Sizes.

Figures 13, 14, 15, 16 show the amount of video distortion, dependency distortion, ISD and EED, respectively, with 95% confidence interval for different number of peers and block sizes ([128,512,1024]) when the third scenario is considered for peer churning. Here, the amount of *T* is selected randomly between 0.5 and 1 second using normal distribution method and the averaged lifetime duration of peers is 300 seconds. In this regard, peers join rapidly but stay for a shorter time in the network. Although in this scenario the behavior of the network is more dynamic, similar to the scenarios 1 and 2, the MATIN provides higher video quality with lower end-to-end and initial startup delays. This means that MATIN makes the P2P network more robust in high churning scenarios.

Finally, it is necessary to be pointed out that the bigger range of the confidence interval in scenarios 2 and 3 is due to churning event. In other words, different runs of the simulation in different seeds cause video distortions with larger differences in their amounts in comparison with the first scenario. Following Figures 5, 6, 7, 8, 9, 10, 11, 12, 13, 14, 15, 16, Table 6 compares MATIN and RNC-GJE in all scenarios numerically.

#### B.2.4 Complementary Discussion

**Figure 17:**
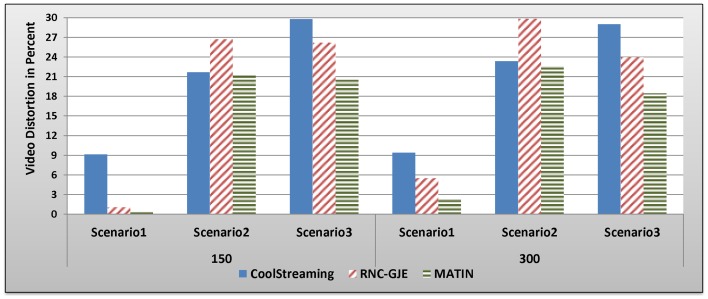
Averaged amounts of Video Distortions in Percent Using CoolStreaming, RNC-GJE and MATIN.

Although previous studies such as [37] show that the perceived video quality on peers can be considerably increased using RNC, in order to confirm this assertion, we compare the MATIN and the RNC-GJE with one of the most popular live video streaming systems, named the CoolStreaming [40], in the same network conditions. According to this fact that high video quality is the target of all video streaming systems, this study compare the introduced amounts of video distortion using these methods as it is depicted in Figure 17. The results show that the MATIN considerably outperforms others. In Figure 17, the averaged amounts of the video distortion in the RNC-GJE and MATIN are depicted based on the different block sizes. In Scenario 2, the RNC-GJE averagely introduces more video distortion due to high transmission overhead. Therefore, existing challenges in RNC can decrease its performance in some special situations so that other methods, which do not employ network coding, outperform the RNC-GJE. However, the MATIN averagely provides a bit better video quality than that of the CoolStreaming in the second scenario. It means that the MATIN efficiently addresses existing challenges in the RNC.

In previous sections, we showed that employing the Gauss-Jordan elimination method in decoders causes some problems such as high transmission overhead and computational complexity. The Gauss-Jordan elimination method needs to have the whole coefficients of a coefficients vector *C_i_* for decoding the related encoded block *X_i_*. This is why the RNC-GJE imposes considerable transmission overhead on the network which leads to higher video distortion. Moreover, according to Figure 4, the Gauss-Jordan elimination method imposes very high computational complexity on a peer in decoding. Based upon the obtained results in section 5, we believe that the MATIN can be a possible answer to the big question propounded in this study. It not only imposes very low transmission overhead and computational complexity on the system, but also provides better video quality on peers and decreases the amounts of end-to-end and initial startup delays, even if the network size increases and churning occurs in the system. Finally, Table 7 compares the MATIN with the RNC-GJE in summary.

**Table 7:** Comparison between MATIN and RNC-GJE in Summary.

Subject	MATIN	RNC-GJE
Transmission Overhead	Very Low	High
Number of Selected Random Values for Generating C_n×n_	*n*	*n^2^*
Computational Complexity	Very Low	Very High
Progressively Decoding	Yes	Yes
Linear Dependency in C^−1^_n×n_	Not Exist (No need to check)	It is necessary to check
Provided Video Quality	High	Lower than that of MATIN
Robust in Peer Churning	High	Lower than that of MATIN

## Conclusion

Recent studies have shown that better perceived video quality can be provided on peers using RNC. However, this method imposes high transmission overhead and computational complexity on the system as they are mentioned in these studies. The imposed computational complexity is due to large number of arithmetic operations in decoding the encoded blocks and checking linear dependency among the coefficients vectors using the most recently used method, the Gauss-Jordan elimination. In addition, the decoder needs to have all values of a coefficients vector to be able to decode the related encoded block which imposes high transmission overhead. This paper introduced MATIN framework in order to efficiently address these challenges. The MATIN generates the required coefficients matrix using *n* instead of *n^2^* entries in Galois Field so that there is no linear dependency among its vectors. Then, it sends one instead of n coefficient entries as the header of each encoded blocks. Therefore, the transmission overhead sharply decreases.

Moreover, it can progressively produce the inverted coefficients matrix using very few numbers of arithmetic operations. As a result, very low computational complexity is imposed on the system. In order to evaluate the performance of the MATIN in P2P live video streaming, we simulated it using a precise simulator in OMNET++ and under different network conditions. Four performance metrics including total video distortion, video distortion due to frame dependency, end-to-end delay (EED) and initial startup delay (ISD) are considered. This study also considered three different scenarios for churning. In the first scenario no churn happened, whereas the second and the third scenarios were based upon two different churning rates and peers' lifetimes.

The results showed that the MATIN considerably outperforms the RNC-GJE, which employs the Gauss-Jordan elimination method in decoding, in terms of the four considered performance metrics in all scenarios. In fact, it provides higher video quality by introducing lesser video distortion and delivered video packets in lower end-to-end delay. Finally, peers finished their initial buffer time earlier. In this regard, the MATIN can be a possible answer to the propounded big question in this study and permits RNC to be more efficient in data multicasting, especially video streaming. In future studies, we aim to implement the MATIN framework over the PlanetLab for evaluating its performance in a real-world P2P network.

## References

[1] Tarkoma S (2010) Overlay Networks: Taylor and Francis group.

[2] Brogle M, Milic D, Bettosini L, Braun T (2009) A performance comparison of native IP Multicast and IP Multicast tunneled through a Peer-to-Peer overlay network; 2009 12–14 Oct. 1–6.

[3] Buford JF, Yu H, Lua EK (2009) Chapter 8 – Peer-to-Peer Content Delivery. P2P Networking and Applications. Boston: Morgan Kaufmann. 183–202.

[4] Picconi F, Massaoulie L (2008) Is there a future for mesh-based live video streaming. IEEE Computer Society, 8th International conference on peer-to-peer computing.

[5] Magharei N, Rejaie R, Yang G (2007) Mesh or Multiple-Tree: A Comparative Study of Live P2P Streaming Approaches; 2007 6–12 May. 1424–1432.

[6] Banerjee S, Bhattacharjee B, Kommareddy C (2002) Scalable application layer multicast. SIGCOMM Comput Commun Rev 32: 205–217.

[7] Magharei N, Rejaie R (2009) PRIME: peer-to-peer receiver-driven mesh-based streaming. IEEE/ACM Trans Netw 17: 1052–1065.

[8] Meng Z, Qian Z, Lifeng S, Shiqiang Y (2007) Understanding the Power of Pull-Based Streaming Protocol: Can We Do Better? Selected Areas in Communications, IEEE Journal on 25: 1678–1694.

[9] Ramzan N, Park H, Izquierdo E (2012) Video streaming over P2P networks: Challenges and opportunities. Signal Processing: Image Communication 27: 401–411.

[10] Xinyan Z, Jiangchuan L, Bo L, Yum YSP (2005) CoolStreaming/DONet: a data-driven overlay network for peer-to-peer live media streaming; 2005 13–17 March. 2102–2111 vol. 2103.

[11] SopCast (2007) Streaming over Peer-to-Peer, Available: http://www.sopcast.com.

[12] Wang M, Li B (2007) R2: Random Push with Random Network Coding in Live Peer-to-Peer Streaming. Selected Areas in Communications, IEEE Journal on 25: 1655–1666.

[13] Ho T, Lun DS (2008) Network Coding: An Introduction. Cambridge, U.K: Cambridge University Press.

[14] Noh J, Girod B (2012) Robust mobile video streaming in a peer-to-peer system. Signal Processing: Image Communication 27: 532–544.

[15] Richardson IE, editor (2010) The H.264 advanced video compression standard.

[16] Baochun L, Di N (2011) Random Network Coding in Peer-to-Peer Networks: From Theory to Practice. Proceedings of the IEEE 99: 513–523.

[17] Heide J, Pedersen MV, Fitzek FHP, Larsen T (2012) Chapter 4 – Network Coding in the Real World. In: Muriel M, Alex S, editors. Network Coding. Boston: Academic Press. 87–114.

[18] Barekatain B, Aizaini Maarof M, Ghaeini HR (2012) The Efficiencies of Decoding Methods in Random Network Coding for Video Streaming. Journal of Basic and Applied Scientific Research 2: 6588–6595.

[19] Ahlswede R, NC, Li S-YR, Yeung RW (2000) Network information flow. Information Theory, IEEE Transactions on 46: 1204 – 1216.

[20] Qureshi J, Foh CH, Cai J (2012) Optimal Solution for the Index Coding Problem Using Network Coding over GF(2). Sensor, Mesh and Ad Hoc Communications and Networks (SECON), 9th Annual IEEE Communications Society Conference on. Seoul. 209–217.

[21] Tabatabaii HSA, Khansari M, Rabiee HR (2010) LiveCod: A mesh-pull P2P live streaming system with XOR-based Network Coding; 2010 6–10 Dec. 436–441.

[22] Katti S, Rahul H, Hu W, Katabi D, Muriel M, et al (2006) XORs in the air: practical wireless network coding. SIGCOMM Comput Commun Rev 36: 243–254.

[23] Ho T, Koetter R, Médard M, Karger DR, Effros M (2003) The Benefits of Coding over Routing in a Randomized Setting. IEEE International Symposium on Information Theory.

[24] Wang M, Baochun L (2007) Lava: A Reality Check of Network Coding in Peer-to-Peer Live Streaming; 2007 6–12 May. 1082–1090.

[25] Anh N, Baochun L, Eliassen F (2010) Chameleon: Adaptive Peer-to-Peer Streaming with Network Coding; 2010 14–19 March. 1–9.

[26] Cleju N, Thomas N, Frossard P (2011) Selection of network coding nodes for minimal playback delay in streaming overlays. Multimedia (csMM); Information Theory (csIT); Networking and Internet Architecture (csNI). Cornell University: IEEE Transactions on Multimedia.

[27] Xiao S, Wang H, Wu C (2011) A new network coding design for reliable video multicast. Chinese Journal of Electronics 20: 361–364.

[28] Jingjing S, Bojin Z, Anni C, Yinbo C (2009) Layered Network Coding and Hierarchical Network Coding for Peer-to-Peer Streaming; 2009 16–17 May. 139–142.

[29] MacKay DJC (2005) Information Theory, Inference, and Learning Algorithms: Cambridge University Press.

[30] Dickson LE (2007) Linear Groups with an Exposition of Galois Field Theory: Cosimo Classics.

[31] Bretscher O (2009) Linear Algebra with Application: Library of Congress Cataloging-in-Publication Data.

[32] Shojania H, Baochun L (2009) Pushing the Envelope: Extreme Network Coding on the GPU. Distributed Computing Systems, 2009 ICDCS '09 29th IEEE International Conference on. Montreal, QC. 490–499.

[33] Keller L, Le A, Cici B, Seferoglu H, Fragouli C, et al.. (2012) MicroCast: cooperative video streaming on smartphones. Proceedings of the 10th international conference on Mobile systems, applications, and services. Low Wood Bay, Lake District, UK: ACM. 57–70.

[34] Li Y, Soljanin E, Spasojevic P (2011) Effects of the Generation Size and Overlap on Throughput and Complexity in Randomized Linear Network Coding. Information Theory, IEEE Transactions on 57: 1111–1123.

[35] Hoffman K, Kunze R (1971) Linear Algebra; Second, editor: Prentice-Hall.

[36] Fragouli C, Soljanin E (2007) Network Coding Applications: now published Inc.

[37] Mirshokraie S, Hefeeda M (2010) Live Peer-to-Peer Streaming with Scalable Video Coding and Network Coding. In Proc of ACM Multimedia Systems (MMSys'10). Scottsdale. 123–132.

[38] OMNET++ (2010) Objective Modular Network Testbed in C++, Avaialble: http://www.OMNETPP.org.

[39] Arizona ASU (2009) Video Traces Research Group on MPEG-4.

[40] Xie S, Li B, Keung GY, Xinyan Z (2007) Coolstreaming: Design, Theory, and Practice. Multimedia, IEEE Transactions on Multimedia 9: 1661–1671.

